# Supplementary material for: Abnormal Alpha-1 Antitrypsin Levels and Other Risk Factors Associated with Lung Function Impairment at 6 and 12 Months after Hospitalization Due to COVID-19: A Cohort Study
Source: Healthcare (Basel). 2022 Nov 22;10(12):2341. doi: 10.3390/healthcare10122341 (PMC9777719; doi:10.3390/healthcare10122341)
Supplement: Supplementary file 1 [file healthcare-10-02341-s001.zip › healthcare-2000040-supplementary.pdf]

Supplementary Tables

Table S1. Summary of the characteristics of the patients included in the study

**First follow-up consultations**
Goes for consultation, N=182 patients
Characteristics
Age (years), median (IQR)
Sex, N (%):
Male
Female
BMI (kg/m2)
Smoking history, N° (%):
Cumulative tobacco burden index **(**ICAT**)**, N° (%):
Current
Former
Nonsmoker
Personal history.
Baseline comorbidities <2; ≥2, N° (%)
High blood pressure (HBP), N° (%)
Diabetes Mellitus 2 (DM2), N° (%)
Dyslipemia, N° (%)
Ischemic cardiopathy: N° (%)
Cardiac arrhythmias N° (%)
Cerebrovascular disease, N° (%)
Baseline respiratory comorbidities, N° (%)
Chronic obstructive pulmonary disease (COPD), N° (%)
Baseline liver disease, N° (%)
Chronic kidney disease, N° (%)

Active tumor, N° (%)
History of previous tumor <5 años, N° (%)
Charlson Comorbility index <3; ≥3, N° (%)
Admission symptoms
Fever (>37.5ºC), N° (%)
Dyspnea, N° (%)
Cough, N° (%)
Fatigue, N° (%)
Musculoskeletal discomfort, N° (%)
Anosmia/ageusia, N° (%)
Digestive symptoms, N° (%)
Cardiac symptoms, N° (%)
Neurological symptoms, N° (%)
Dermatological symptoms, N° (%)
ARDS (PaO2/FIO2 < 300 mmHg):
Hospitalization.
Average length of stay (days)
Pneumonia on admission No. (%)
• Characteristics of pneumonia on chest X-ray, No. (%)<br>-Density:<br>Alveolar<br>Ground glass opacity (GGO)<br>Mixed<br>-Distribution:<br>Central<br>Peripheral<br>Mixed<br>-Location:<br>Unilateral<br>Bilateral<br>-Extension:<br>Unilobar<br>Multilobar

ICU admission, No. (%)
Orotracheal intubation (OTI, No. (%)
Treatment received, No. (%)<br>- Corticosteroids<br>- Antiviral drugs<br>- Tocilizumab<br>- Anakinra<br>- Antimalarials (hydroxychloroquine)
**Second follow-up consultations**
Goes for consultation, N= 168
Pulmonary function
FVC <80%, % of predicted
FEV1 <80%, % of predicted
FEV1/FVC <70%
TLC <80%, % of predicted
VR <65%, % of predicted
DLCO <80%, % of predicted
KCO<80%, % of predicted
6MWT
Distance-meters, median (IQR)
Oxygen saturation, median (IQR)
Average
Initial
Final
**Third follow-up consultations**
Goes for consultation, N=157
Pulmonary function performed only on patients with alterations at 6 months.
Laboratory findings at 12 months (mean ± SD)
Alpha 1 antitrypsin (mg/dl)
Alpha antitrypsin normal range, N° (%)

ARDS=Acute respiratory distress syndrome; ICU= Intensive care unit; BMI= body mass index; IOT=Intubation orotraqueal; FVC=forced vital capacity; FEV1=forced expiratory volume in the first second; TLC=total lung capacity; RV=residual volume; DLCO=carbon monoxide transfer by single breath; KCO= diffusion constant for carbon monoxide; 6MWT=six-minute walking test; SD: standard deviation

Table S2. Laboratory parameters of patients on admission, during hospitalization and at the 12-month follow-up

**Laboratory parameters**	**Lung function at 12 months**		**p-value**
	**Normal (n=108) (mean ± SD) or n (%)**	**Impaired (n=49) (mean ± SD) or n (%)**	
**On admission**			
Hemoglobin (g/dl)	14.5±1.5	13.7±2.1	0.017
Leukocytes (count x10^3/µl)	6.3±2.6	7.7±3.2	0.011
Neutrophils (count x10^3/µl)	4.8±2.4	5.9±2.7	0.024
Lymphocytes (count x10^3/µl)	1.3±1.1	1.1±0.5	0.044
Ferritin (ng/ml)	745.3±857.9	624.5±688.3	0.523
Platelets (count/µl)	214,819±85,918	263,851±135,238	0.012
C-Reactive Protein (mg/l)	79.2±68.8	88.7±73.1	0.358
Lactate dehydrogenase (U/l)	344.1±122.5	372.4±113.3	0.161
AST/Aspartate transaminase (U/l)	44.0±31.8	38.2±26.3	0.407
ALT/Alanine transaminase (U/l)	40.0±30.1	32.9±22.2	0.736
Albumin (g/dl)	4.0±0.8	5.1±6.9	0.360
D-dimer (mg/l)	0.76±1.07	2.3±5.4	0.084
Fibrinogen (mg/dl)	641.0±193.7	666.6±213.2	0.255
Blood glucose (mg/dl)	115.2±38.2	123.8±55.0	0.307
**During hospitalization**			
Hemoglobin (g/dl)	15.4±15.9	13.0±2.0	0.008
Leukocytes (count x10^3/µl)	7.6±3.7	8.0±3.1	0.466

Neutrophils (count x10³/μl)	6.1±5.1	6.0±3.3	0.969
Lymphocytes (count x10³/μl)	1.4±1.0	1.3±0.8	0.206
Ferritin (ng/ml)	1,133±1,560	1,577±2,043	0.197
Platelets (count/μl)	323,641±188,349	329,674±163,188	0.993
C-Reactive Protein (mg/l)	54.2±102.5	70.3±77.2	0.400
Lactate dehydrogenase (U/l)	328.8±104.3	386.6±145.2	0.011
AST/Aspartate transaminase (U/l)	40.4±34.0	58.5±64.7	0.213
ALT/Alanine transaminase (U/l)	64.8±118.3	99.3±163.4	0.078
Albumin (g/dl)	3.8±0.5	3.6±0.5	0.048
D-dimer (mg/l)	2.1±8.3	2.8±5.4	0.662
Fibrinogen (mg/dl)	589.8±223.5	590.7±253.4	0.938
Blood glucose (mg/dl)	112.1±48.8	135.6±87.4	0.061
IL-6 (pg/ml)	20.5±4.4	57.1±13.5	0.090
**At 12-month follow-up**			
Hemoglobin (g/dl)	15.0±1.5	14.2±2.0	0.012
Leukocytes (count x10³/μl)	6.6±1.8	7.0±1.5	0.633
Neutrophils (count x10³/μl)	4.0±2.6	4.3±2.9	0.995
Lymphocytes (count x10³/μl)	2.2±0.9	2.2±0.8	0.536
Ferritin (ng/ml)	173.3±364.5	91.8±76.8	0.023
Platelets (count/μl)	221,820±61,508	256,583±113,230	0.028
C-Reactive Protein (mg/l)	3.5±4.6	4.6±5.6	0.358
Lactate dehydrogenase (U/l)	179.73±34.6	200.3±60.0	0.017
AST/Aspartate transaminase (U/l)	25.8±15.6	22.4±7.5	0.261

ALT/Alanine transaminase (U/l)	28.6±28.0	22.3±13.0	0.134
Albumin (g/dl)	4.7±4.3	4.2±0.3	0.442
D-dimer (mg/l)	0.5±0.6	0.8±1.9	0.217
Fibrinogen (mg/dl)	371.4±435.2	380.9±181.3	0.856
Blood glucose (mg/dl)	97.1±20.5	106.6±41.4	0.091
IL-6 (pg/ml)	6.4±12.8	4.9±10.3	0.413
Vitamin D (ng/ml)	22.7±8.7	19.5±9.2	0.042
Phosphorus (mg/dl)	3.3±1.1	3.4±1.1	0.790
Calcium (mg/dl)	9.5±1.1	9.3±1.0	0.472
Alpha-1 antitrypsin (mg/dl)	124.3±15.4	129.1±18.2	0.480
Alpha antitrypsin range abnormal	1 (1.1)	1 (1.1%)	0.166
Serology, IgG Ab +ve	98 (95.1)	43 (91.5)	0.536

SD: standard deviation

Tabla S3. Number and proportion of hospitalized patients with normal or impaired lung function at 12 months with plasma determination of alpha-1 antitrypsin (AAT), allelic genotyping (Pi*MM, Pi*MS, Pi*MZ and Pi*MP Lowell) and C-reactive protein (CRP) >5 mg/dl.

Genotype	Lung function at 12 months						p-value
	Normal (n=108)			Impaired (n=49)			
	N (%)	AAT mean ± SD	CRP >5 N (%)	N (%)	AAT mean ± SD	CRP >5 N (%)	
Pi*MM	91 (84.2)	126.4±14.7	15 (20)	40 (81.6)	133.9±14.4	9 (18.4)	0.016
Pi*MS	16 (14.8)	116.3±15.4	5 (4.6)	7 (14.2)	116.7±15.4	0 (0)	0.962
Pi*MZ	0	-	0	2	89.0±21.2	0 (0)	-
Pi*MP Lowell	1 (0.9)	95.0	0	0	-	0	-

SD: standard deviation

Table S4. Results of multiple comparison tests between different alpha-1 antitrypsin (AAT) genotyping variants and respiratory function parameters at 6 months.

Parameter	Genotype		Mean difference	SD	p-value
FEV1 (%)	Pi*MM	Pi*MS	8.4	4.4	0.176
		Pi*MZ	21.6	12.3	0.319
		Pi*MP	-	-	-
	Pi*MS	Lowell	13.2	13.8	1.000
		Pi*MZ			
FVC	Pi*MM	Pi*MS	-33.1	16.4	0.139
		Pi*MZ	14.7	49.7	1.000
		Pi*MP	-	-	-
	Pi*MS	Lowell	47.8	51.6	1.000
		Pi*MZ			
FEV1/FVC	Pi*MM	Pi*MS	0.97	2.1	1.000
		Pi*MZ	8.2	6.0	0.508
		Pi*MP	-	-	-
	Pi*MS	Lowell	7.3	6.2	0.728
		Pi*MZ			
TLC %	Pi*MM	Pi*MS	0.93	3.5	1.000
		Pi*MZ	28.7	10.6	0.023
		Pi*MP	-	-	-
	Pi*MS	Lowell	27.8	11.0	0.039
		Pi*MZ			
DLCO %	Pi*MM	Pi*MS	4.3	5.2	1.000
		Pi*MZ	41.9	15.5	0.024
		Pi*MP	-	-	-
	Pi*MS	Lowell	37.5	16.1	0.064
		Pi*MZ			
KCO %	Pi*MM	Pi*MS	-0.21	4.3	1.000
		Pi*MZ	20.5	12.8	0.335
		Pi*MP	-	-	-
	Pi*MS	Lowell	20.7	13.3	0.364

		Pi*MZ			
RV %	Pi*MM	Pi*MS	-13.8	6.4	0.099
		Pi*MZ	30.9	18.9	0.316
		Pi*MP	-	-	-
	Pi*MS	Lowell Pi*MZ	44.7	19.7	0.075
6MWT Distance (m)	Pi*MM	Pi*MS	21.7	24.9	1.000
		Pi*MZ	210.2	74.9	0.017
		Pi*MP	-	-	-
	Pi*MS	Lowell Pi*MZ	188.5	77.7	0.050
6MWT Initial SaO2 (%)	Pi*MM	Pi*MS	0.02	0.38	1.000
		Pi*MZ	1.6	1.14	0.492
		Pi*MP	-	-	
	Pi*MS	Lowell Pi*MZ	1.6	1.2	0.558
6MWT Final SaO2 (%)	Pi*MM	Pi*MS	-0.01	0.88	1.000
		Pi*MZ	5.5	2.6	0.140
		Pi*MP	-	-	-
	Pi*MS	Lowell Pi*MZ	5.5	2.7	0.118

FVC=forced vital capacity; FEV1=forced expiratory volume in the first second; TLC=total lung capacity; RV=residual volume; DLCO=diffusing capacity for carbon monoxide; 6MWT=six minute walk test; SaO2=oxygen saturation; SD= standard deviation
